# Adaptive AI-based mobile simulation for drug calculation competency in nurses: a mixed-methods RCT

**DOI:** 10.1186/s12912-026-05313-4

**Published:** 2026-09-08

**Authors:** Mohamed Fakhry Ahmed Salem, Ahmed Khamis Sharaf, Ahmed abdelhafeez abdelmonseif Younis

**Affiliations:** 1 Medical-Surgical Nursing Department, Faculty of Nursing, Alexandria University, Alexandria, Egypt; 2 Community Health Nursing Department, Faculty of Nursing, Alexandria University, Alexandria, Egypt; 3 Critical Care Nursing Department, Faculty of Nursing, Alexandria University, Alexandria, Egypt

**Keywords:** Artificial intelligence, Nursing education, Drug dose calculation, Clinical decision-making, Self-efficacy, Medication safety

## Abstract

**Background:**

Medication dose miscalculations remain one of the most persistent patient safety concerns in clinical practice, while traditional teaching methods often fall short in ensuring proficiency. The integration of artificial intelligence (AI) based learning tools has the potential to enhance knowledge, clinical decision-making (CDM), and self-efficacy among nursing staff.

**Aim:**

To evaluate the effectiveness of a researcher-developed artificial intelligence-based mobile learning application designed to simulate realistic hospital medication environments with adaptive feedback on nursing’s drug calculation knowledge, decision-making, and self-efficacy, while exploring its feasibility and acceptability in routine training contexts.

**Design:**

Mixed-methods study with an embedded design, utilizing two-arm randomized controlled trial for quantitative evaluation and focus group discussions for qualitative exploration.

**Methods:**

The study was conducted at a non-governmental hospital where 56 nurses, randomly assigned to intervention group (*n* = 28) or a control group (*n* = 28). The mobile learning application simulated realistic hospital medication administration scenarios incorporating adaptive corrective feedback and progressively complex drug calculation cases. Data was collected at baseline and post-intervention using a drug dose calculation knowledge assessment, a clinical decision-making scale, and a self-efficacy scale. Post-intervention focus group discussions with the intervention group were analyzed using thematic analysis.

**Results:**

The study group achieved significant improvements in knowledge, decision-making, and self-efficacy post-intervention compared to the control group with large effect sizes. Qualitatively, six themes emerged which discussed several aspects of the training such as; perceived clinical benefits, realism, increased nurses’ confidence, ease of use and engagement, training efficiency and challenges and the potential for improvement.

**Conclusion:**

This is one of the first randomized controlled trials (RCTs) conducted using mixed-methods and focusing on training nursing staff on drug-dose calculation with artificial intelligence (AI) based simulation. Results indicated that the intervention was associated with significant improvements in the measured outcomes and was perceived positively by the participants. However, its broader implementation across different contexts requires further investigation.

**Trial registration:**

ClinicalTrials.gov Identifier: NCT07448259. Retrospectively registered on February 18, 2026. Registration Link: https://clinicaltrials.gov/study/NCT07448259.

**Supplementary Information:**

The online version contains supplementary material available at https://doi.org/10.1186/s12912-026-05313-4.

## Impact

Manual drug-dose calculation errors and delayed decision-making have been identified as a significant contributors of patient safety risks.

Artificial intelligence-based mobile learning intervention improves nurses' drug calculationaccuracy, decision-making speed, and self-efficacy compared to traditional methods.

These findings highlight the need for future studies to evaluate the scalability of this training approach and its long-term impact on patient safety and care quality.

**Supplementary Information:**

The online version contains supplementary material available at https://doi.org/10.1186/s12912-026-05313-4.

## Introduction

Medication dose miscalculations remain one of the most persistent patient safety concerns in clinical practice, and recent studies have demonstrated that up to 70% of nurses have been involved in or witnessed medication dose errors, highlighting the paramount importance of enhanced numeracy and calculation skills in clinical practice [1]. Systematic reviews also indicate that over half of nursing students around the globe fail drug calculation tests. Knowledge gaps, conceptual misconceptions, and anxiety are among the major contributors to unsafe practice [2]. These findings highlight an urgent need for novel educational models that can enhance accuracy, clinical thinking, and decision-making confidence regarding drug dosages. The present study is theoretically based on Kolb’s Experiential Learning Theory and Bandura’s Self-Efficacy Theory, which together provide a framework for the use of simulated environments to develop clinical competence through active, reflective, and adaptable learning experiences [3, 4].

Artificial Intelligence (AI)-based learning has emerged as a promising intervention to enhance medication calculation competence due to the rapid digitalization of healthcare. Simulations involving AI have been demonstrated to enhance communication, clinical reasoning, knowledge acquisition, and self-efficacy through adaptive, personalized, real-time feedback mechanisms [5]. Despite the increasing use of AI in nursing education, several gaps remain unexplored. Most intervention studies focused on students’ education rather than practicing nurses’ professional development. They also mainly examined the impact on knowledge without addressing the impact on clinical decision-making and self-efficacy. In addition, few studies used rigorous RCT designs combined with qualitative exploration specifically in resource-limited settings. Therefore, this study aims to address these gaps by evaluating an AI-based application that improves multiple competencies among nursing staff.

## Background

AI-enhanced virtual reality promotes iterative decision-making, psychological safety, accelerated skill acquisition and outperforms traditional simulation across several experiential learning dimensions [6]. Such environments enable nursing staff to practice complex dosage calculations repeatedly, without the cognitive demands of real clinical settings [1]. A comprehensive umbrella review confirms that artificial intelligence enhances decision-making, workflow operations, and evidence-based practice, while highlighting the need to enhance transparency, ethical protection, and faculty willingness to achieve responsible application in the teaching of nursing staff [7]. Qualitative evidence indicates that artificial intelligence-guided dosing can enhance accuracy, decrease medication errors, and allow personalized dosing, but they need to be understandable, and clinicians must retain oversight to prevent over-reliance [8].

In addition to AI-specific systems, digital learning methods, such as web-based training, gamified learning, and digital physical reality, are consistently demonstrating improved conceptual knowledge and psychomotor accuracy, as well as decreased medication calculation errors due to immersive and interactive experiences [9]. Screen-based simulations have also shown significant improvements in the dosage calculation scores and confidence of students after structured interventions of virtual training [10]. On the system level, AI was proven to increase the clarity of medication instruction and minimize the risks associated with prescription [11]. Other applications of AI to nursing roles, assisting patients, and improving clinical decision making are also seen in reviews that must be addressed using well-organized frameworks of safe implementation [12].

The current mobile learning application uniquely integrates adaptive clinical environment simulation, targeting the simultaneous growth of knowledge, clinical decision-making, and self-efficacy, unlike many existing digital learning interventions that primarily focus on knowledge acquisition. Despite the growing body of evidence supporting artificial intelligence-based learning, significant methodological and contextual gaps remain focus on pre-registration nursing staff and have been conducted exclusively in high-income contexts. To the best of our knowledge, few studies have examined AI-based adaptive simulation aimed at simultaneously improving drug calculation knowledge, clinical decision-making, and self-efficacy among qualified nursing staff, particularly in resource-limited settings where facilities for medication management training are scarce, such as in Egypt. In such a healthcare context, challenges such as limited access to standardized training, variability in clinical skill development, and reliance on traditional teaching methods may contribute to medication errors. These factors make Egypt a relevant setting for evaluating scalable, technology-enhanced training approaches. This ultimately addresses the World Health Organization’s global call for innovative, scalable solutions to improve nurse competence and reduce medication errors [13].

Overall, current evidence suggests that AI-based and digital learning approaches can improve medication-related knowledge, clinical reasoning, and learner engagement. However, these positive outcomes were inconsistently reported across different contexts, and research gaps regarding its potential application in resource-poor settings and with practice nurses remain unexplored. To address these gaps, this study aimed to assess the effectiveness of a researcher-developed mobile learning application designed to create realistic hospital medication administration situations on medication knowledge, clinical decision-making, self-efficacy beliefs, and to investigate the feasibility and acceptability of using the application in in-service training of nurses. To date, this is one of the first randomized controlled trials to investigate such an intervention with trained clinical nursing personnel in a resource-constrained setting.

## Study aim, objectives, and hypotheses

This study aimed to evaluate the effectiveness of a researcher-developed artificial intelligence-based mobile learning application specifically designed to simulate realistic hospital medication administration environments on nursing staff’s drug-dose calculation knowledge, clinical decision-making, and self-efficacy, while exploring its feasibility and acceptability within routine training contexts. All quantitative outcomes (knowledge, clinical decision-making, and self-efficacy) were assessed at baseline and at two weeks post-intervention.

### Primary objective

To evaluate the effectiveness of an artificial intelligence-based mobile learning application compared to traditional instruction on nursing staff’s drug-dose calculation knowledge at two weeks post-intervention.

### Secondary objectives

- To compare post-intervention scores of clinical decision-making and self-efficacy between the AI-based learning group and the traditional group.
- To explore intervention feasibility and acceptability by nurses through focus group discussions to provide an in-depth understanding of participants’ experiences and to complement the quantitative findings.

### Hypotheses

#### H1

Nurses receiving AI-based learning will demonstrate significantly higher post-intervention scores in drug-dose calculation knowledge, as well as improved clinical decision-making and self-efficacy, compared to those receiving traditional methods.

## Methods

### Design

This study employed an embedded mixed-methods design, utilizing a two-arm randomized controlled trial (RCT) design for the quantitative component and focus group discussions embedded within the intervention group to assess the feasibility and acceptability of the artificial intelligence-based mobile learning application. The qualitative strand was essential to explain the mechanisms underlying quantitative outcomes. The quantitative component was prioritized to evaluate the effectiveness of the intervention, while the qualitative data were collected post-intervention to provide explanatory insights into participants’ experiences and to complement the quantitative findings. Participants were randomized after baseline assessment into intervention and control groups. Integration of quantitative and qualitative findings was achieved during the interpretation phase, where qualitative results were used to explain and contextualize the quantitative outcomes (Supplementary materials II).

### Study setting and sampling

Participants were recruited from a non-governmental hospital in Egypt, comprising approximately 120 beds and offering a large variety of services, including general wards, emergencies, critical care, coronary care, and home-care nursing. This setting was selected for its varied clinical environment and established in-service training infrastructure. 

#### Sampling and randomization

The eligible nurses were enrolled in the hospital’s in-service training program and then randomized to 2 equal groups of 28 nurses each. Permuted block randomization with a block size of 4 was employed to allocate participants (1:1) equally to intervention and control groups. Stratification by clinical sectors ensured balanced exposure to medication-related tasks. Clinical sectors were operationally defined based on participants assigned working units (e.g., medical-surgical wards, ICU, ER, CCU). Balance across strata was verified by comparing the distribution of participants across clinical sectors between groups after randomization, confirming no significant differences. Allocation concealment was maintained using consecutively numbered, opaque, sealed envelopes (SNOSE), prepared by an independent administrator not involved in the recruitment or study implementation.

#### Sample size calculation

A priori power analysis using G*Power 3.1, based on an expected medium-to-large effect size of 0.68 [14] alpha of 0.05, and power of 0.80 for a two-tailed independent samples t-test, indicated a required sample of 56 participants (28 per group). Recruitment, allocation, and follow-up were documented according to the CONSORT 2010 statement for randomized controlled trials to ensure transparency and reproducibility.

### Inclusion and exclusion criteria

**Inclusion Criteria**: Nurses who were directly involved in patient care across various clinical units where medication administration and drug dosage calculations are routinely performed as part of their daily clinical duties, and available to participate throughout the training period. **Exclusion Criteria**: Nurses who have recently received drug-calculation training (within the past 6 months) or had prior exposure to artificial intelligence-based educational tools (within the last 6 months) to minimize baseline differences in knowledge and prior exposure that could confound.

### Study intervention

The intervention involved a three-session structured training delivered through a researcher-developed AI-based drug calculation mobile application that enhanced learning through interactive, case-based scenarios covering oral medication calculations, intravenous infusion adjustments, and pediatric dosing.

Both groups were taught based on identical learning outcomes, but the mode of delivery would be different. The control group was taught in the traditional face-to-face method through lectures, guided discussion, and paper-based problem-solving exercises with equal contact time confirmed. The traditional teaching sessions were standardized using structured lesson plans that included clearly defined learning objectives, guided explanations, and supervised problem-solving exercises to ensure consistency across sessions. The intervention group received the same content supplemented by AI-based drug-calculation mobile application, where they received personalized feedback and adaptive learning paths. The training was conducted in three sessions carried out in two weeks, with every session taking around 60–90 min.

- **Session 1**: Orientation about the mobile application and explained basic drug-calculation principles, unit conversions, and dosage formulas.
- **Session 2**: Applying interactive medication scenarios such as oral dosage calculations, intravenous infusions, and weight-based adjustments.
- **Session 3**: Advanced practice, including infusion-rate adjustment, high-alert medications, and error detection utilizing high-fidelity AI-generated clinical scenarios.

### Description of AI-powered clinical drug calculation mobile learning application

The AI-Powered Clinical Drug Calculation application was developed by one of the authors. It is an evidence-based educational system that was developed in accordance with global medication safety guidelines from the Institute for Safe Medication Practices and the World Health Organization. It provides dynamic, case-based scenarios that mimic real clinical settings by using AI adaptive learning algorithm driven by rule-based logic. In this application, each scenario includes comprehensive patient profiles, prescriptions, lab results, medication concentrations, and formulation variables. Learners interpret clinical data, calculating suitable dosages for oral drugs, intravenous boluses, infusion rates, and weight-based dosing, and evaluate medication appropriateness in a practical setting.

The adaptive engine continuously evaluates learner performance and modifies scenario difficulty based on the user’s real-time performance and accuracy. Adaptation was triggered using predefined performance criteria, including achieving high accuracy thresholds, repeated error patterns, and response time efficiency, which prompted either progression to more complex scenarios or provision of additional practice with targeted feedback. It focuses on clinical decision-making and reflective reasoning through high-fidelity simulations, where nurses control virtual charts, infusion pumps, and track patient reactions. Furthermore, it creates comprehensive performance analytics, such as repeated errors, accurate rate calculation, and response times, to personalize the learning pathway and enhance skill acquisition. This approach enables adaptive, performance-driven learning rather than static repetition of practice problems.

The app’s architecture explicitly integrates Kolb’s Experiential Learning and Bandura’s Self-Efficacy theories. Its three-session structure facilitates a transition from concrete experience to active experimentation through AI simulations. Simultaneously, the app builds mastery experiences via scaffolded difficulty levels and provides verbal persuasion through immediate AI feedback. This theory-to-design mapping supports reflective learning processes and enhances learners’ confidence through structured feedback and progressive skill development. Prompting reflective observation transformed the digital tool into a robust pedagogical intervention that systematically elevates both clinical competence and professional confidence. The application reflects Kolb’s experiential learning cycle by providing concrete clinical scenarios (experience), immediate feedback (reflection), and repeated adaptive practice (active experimentation). Bandura’s self-efficacy theory was emphasized through progressive difficulty levels, mastery experiences, and real-time feedback that would enhance learners’ confidence in performing drug calculations.

#### Fidelity of intervention

All training materials and session plans were reviewed by a panel of five external experts based on predefined criteria, including content relevance, clinical accuracy, and instructional clarity, using a structured review process to reach consensus on the appropriateness of the training materials. Fidelity of interventions was also evaluated by using structured fidelity checklists that were filled out by an independent observer to guarantee consistency in delivery of interventions across sessions. The fidelity checklist included key domains such as adherence to session duration, completeness of content delivery, facilitator adherence to the protocol, and participant engagement during sessions. Post-test assessments were scored by blinded assessors unaware of group allocation to ensure objectivity. This was achieved by assigning coded identifiers to participants’ data, with no indication of group allocation provided to outcome assessors during scoring.

### Data collection measures

#### Socio‑Demographic and Professional Characteristics Structured Questionnaire

A designed Socio-Demographic and Professional Characteristics Questionnaire was developed to gather baseline data regarding the personal and professional characteristics of the participants such as age, gender, education level, clinical experience years, current clinical unit, prior training in drug-dose calculation, prior experience with AI-based educational resources. Content validity was established through a five-expert panel of specialists in nursing education, clinical practice, and pharmacology review with item-level content validity indices of ≥ 0.80. Pilot testing with 10 nurses confirmed clarity and feasibility, with two-week test-retest reliability demonstrating adequate stability (*r* = 0.82).

#### Tool II: drug calculation knowledge

This tool was developed using a multi-stage systematic method to assess the nursing staff knowledge about drug calculation based on the frameworks provided by Bondre (2025), Dilles et al. (2011), Rai and Devi (2019), and Wright (2007) [15–18]. It contained 16 multiple-choice items that included oral medication calculations, intravenous infusion rates, and weight-related dosage. To ensure content validity, the tool was reviewed by five experts from the fields of nursing education, cardiology, and clinical pharmacology. Key competency domains, such as weight-based dosage, intravenous infusion rates, and oral medication calculations, were mapped from the frameworks that were cited. Additional elements on infusion-rate adjustment were added, and clinical examples were adjusted to reflect local nursing practice. Item development and adaptation were conducted through iterative expert review, and every item was assessed for cultural appropriateness, clinical accuracy, content relevancy, and clarity. Items that were redundant, inadequately related to local practice, or lacking appropriate discrimination during expert assessment were amended or eliminated based on consensus before pilot testing. A total score range of 0 to 16 was stratified into three proficiency levels: Low (0–9; <60%) indicates a deficiency in understanding; Moderate (10–12; 60%–80%) reflects acceptable but incomplete knowledge; and High (13–16; >80%) demonstrates mastery with a content validity index of 0.89. The chosen cut-off points had been selected to correspond with competency-based thresholds that frequently employed in healthcare education [19, 20].

Following expert review, items were pilot tested with a separate group of 10 nurses to assess comprehensibility and psychometric performance. Items with discrimination indices below 0.20 or excessive difficulty were revised or removed, yielding the final 16-item instrument. All items were dichotomously scored (1 = correct, 0 = incorrect), each with a single correct answer. Internal consistency was satisfactory (KR-20 = 0.82), with high two-week test-retest reliability (*r* = 0.86).

#### Tool III: nursing decision‑making questionnaire

This is an 8-item questionnaire created by researchers, based on the conceptual model of the Nursing Decision-Making Instrument (NDMI). The original NDMI was not directly modified into the questionnaire. Instead, eight medication-related items covering clinical judgment, safety verification, information evaluation, and application of drug-calculation results in clinical practice were developed using key decision-making domains in the original instrument. Furthermore, to ensure relevance, clarity, and applicability of medication administration practice, the final items were examined and improved by expert evaluation. The domains of clinical decision making from the original instrument and recent cultural adaptation and psychometric testing by Paiva et al. (2024) [21] informed item development. The items were developed to evaluate the undergraduate nursing students’ medication administration decision-making processes, such as medication accuracy, safety-checking practices, critical thinking and using medication calculation results in the clinical context. The face and content validity were determined by expert opinion prior to data collection.

Responses were scored using a 5-point Likert scale from strongly disagree (1) to strongly agree (5) giving a total score of between 8 and 40, with higher scores reflecting better medication-related decision-making. Since there is no established scoring categories for the researcher developed eight item version, the total scores were scored a priori based on an equal interval approach recommended for scales (DeVellis & Thorpe, 2021). The high level (75% or more of the possible score) was selected as a competency criterion that meets the level of ability expected of safe clinical decision making as outlined by Benner (1984). Exploratory factor analysis revealed a one factor solution accounting for 62% of the variance, with Cronbach’s α equal to 0.87 and test-retest reliability equal to *r* = 0.84. Competency categories were assigned based on individual participant scores rather than group mean values. Therefore, participants with a score ≥ 30 were categorized as a high level of competency regardless the overall group mean.

Exploratory factor analysis (EFA) was conducted to assess construct validity. Sampling adequacy was confirmed, and the data were suitable for factor analysis. A single-factor solution was retained based on eigenvalues greater than one, explaining 62% of the total variance. The factor structure was consistent with the theoretical construct of clinical decision-making. The cut-off points were based on proportional segmentation of the total score range, a commonly used approach in scale interpretation when established benchmarks are not available. This method allows for consistent categorization of performance levels and has been supported in similar studies assessing nursing competencies and clinical decision-making. This approach is consistent with previous research that utilized percentage-based classifications to interpret levels of clinical competence and decision-making performance.

#### Tool IV: drug calculation self‑efficacy scale

This scale was adapted from the Generalized Self‑Efficacy Scale by Schwarzer and Jerusalem (1995) [22] to assess the perceived confidence of nurses about their abilities to do correct drug calculations and manage medication related scenarios. The original items were adapted by modifying the medication dosage calculation scenarios without compromising the conceptual framework of the scale. Although the items were contextually adapted to reflect medication-related scenarios, care was taken to preserve the core construct of general self-efficacy, focusing on individuals’ overall confidence in their ability to cope with challenging tasks. The adaptation was limited to contextual framing without altering the underlying theoretical dimensions of the original scale. This was followed by evaluation of these adapted items with a panel of five experts specialized with nursing education, medical–surgical nursing, and psychometrics. This approach is consistent with previous studies that have adapted general self-efficacy scales to specific professional contexts while maintaining construct integrity. Based on a 4-point Likert scale, scores ranged from 10 to 40, categorized into three belief levels: Low (10–20), Moderate (21–30), and High (30–40) [23, 24]. The tool demonstrated excellent reliability (Cronbach’s α = 0.90) and good test-retest reliability (*r* = 0.88), with confirmatory factor analysis indicating a good model fit (CFI = 0.94, RMSEA = 0.05).

#### Focus group discussions

This tool was conducted to explore qualitative dimensions of the participants’ experiences and perceptions of the AI-driven platform. A purposive sampling approach was used to select participants from the intervention group to ensure representation of diverse clinical units and varying levels of engagement with the application. This approach allowed the inclusion of a range of experiences and perspectives. A total of 28 nurses from the intervention group participated in four focus groups, each group contained 7 participants. The duration of each session lasted around 45 to 60 min moderated by a research team member with experience in qualitative interviewing and nursing education while fostering a supportive environment for sharing both benefits and challenges. All focus group interactions were audio-recorded and structured around six key themes as the following: Perceived Clinical Benefits, Realism & Theory-to-Practice, Confidence & Competence, Ease of Use & Engagement, Efficiency & Resource Saving, and Challenges & Suggestions for improvement. Data collection and analysis were 3 done until achieved thematic saturation with no additional themes or meaningful insights. Data saturation was determined when no new codes or themes emerged during the analysis of successive focus groups, and findings became repetitive across groups. This was confirmed through iterative comparison of coded data and continuous review of emerging themes. Although discussions were guided by predefined thematic areas, open-ended questions and probing techniques were used to allow participants to elaborate freely and introduce new perspectives beyond the predefined themes, supporting the inductive nature of the analysis.

### Data collection

Data was collected at two time points, immediately before the commencement of the training to establish initial competency levels, and two weeks after the completion of the final training session to evaluate the mobile learning application’s effectiveness and the retention of knowledge and skills over time. Assessments were conducted in a controlled classroom setting under standardized conditions, with participants completing paper-based instruments simultaneously to prevent inter-group discussion. All post-tests were scored by blinded assessors who were responsible for scoring the knowledge assessment and examining questionnaire responses prior to data entry for all post-tests. They performed scoring using coded participant identities and were not involved with participant recruiting, intervention delivery, or group assignment. Blinding was implemented by assigning coded identifiers to all participant data, with no indication of group allocation. Outcome assessors were not involved in participant recruitment or intervention delivery and had no access to group assignment information during scoring. All the answers were then computerized and scored by using validated scoring keys of the knowledge test and the clinical decision making scale. To minimize potential testing effects associated with repeated use of the same instruments, a two-week interval was maintained between pre- and post-assessments, reducing the likelihood of recall. Additionally, participants were not provided with feedback on their baseline performance, which further limited practice-related bias.

The artificial intelligence application was accessible exclusively to intervention group participants via individualized login credentials and was unavailable on shared devices or public platforms. Training sessions for both groups were scheduled on separate days to prevent cross-group contamination, and intervention participants were explicitly instructed not to share application content or strategies with control group colleagues until study completion.

### Data analysis

SPSS version 26 was used to analyze data. Descriptive statistics provided summary information on demographic and professional characteristics (means, standard deviations and percentages). The Shapiro-Wilk test was used to determine the normality of the data, and parametric tests were employed for normally distributed outcomes (knowledge, clinical decision-making, and self-efficacy). Independent samples t-tests were initially used to compare unadjusted between-group differences. However, ANCOVA was employed as the primary analytical method to control baseline values and provide adjusted comparisons between groups. The internal consistency of the instruments was checked by Cronbach’s alpha and effect sizes (Cohen’s d) were computed for intervention effects. There were no values missing from the final data set. A p value of < 0.05 was considered statistically significant. Qualitative data processing of focus group discussions was done using inductive thematic analysis according to the six steps of Braun and Clarke: familiarizing oneself with the data, coding, generating themes, reviewing, defining, and reporting. Coding was conducted independently by members of the research team, followed by regular discussions to compare interpretations and ensure consistency. Disagreements were resolved through consensus to enhance the credibility and reliability of the thematic analysis. Data completeness was verified through systematic data screening and validation procedures before analysis. As no missing values were identified, a complete-case analysis approach was applied.

### Ethical approval

The present study was approved by the Institutional Review Board (IRB) of the Ethics Committee of the Faculty of Nursing, Alexandria University (Approval No. IRB00013620) on September 19, 2025, before participant recruitment and commencement of the study procedures. The trial was registered retrospectively at ClinicalTrials.gov at ClinicalTrials.gov (Identifier: NCT07448259) on February 18, 2026; Registration Link: https://clinicaltrials.gov/study/NCT07448259, after the completion of data collection. Although prospective registration is recommended, the study design, outcomes, and analysis plan were predefined before implementation and were not modified during or after data collection. The study protocol and statistical analysis plan were developed before the initiation of data collection. All the study procedures, such as recruitment, randomization, intervention delivery, and data management, were followed in accordance with the ethical principles of the World Medical Association Declaration of Helsinki.

Data collection was performed between September 22 and December 30, 2025. Participants provided written informed consent following detailed explanation of study aims, procedures, and their right to withdraw without consequence. Confidentiality of the data was ensured by using coded identifiers, password locked files, and limiting access to re-identification keys. Conflict of interest from the development of the platform by the study author was addressed by having external validation of the platform by independent experts and blinded assessment of the outcomes throughout.

## Results

A total of 56 participants were assessed for eligibility, of whom 28 nurses for each group were randomized.

Baseline demographic and professional characteristics of both groups are presented in Table 1. The median age was 24.50 years in both the control and study group (*p* = 0.902). The majority of participants were males in both control (53.6%) and study group (64.3%) (*P* = 0.415). All participants held a bachelor’s degree in nursing and were employed as staff nurses. A median nursing experience 2 years ranged from 1 to 3 years both the control and study group (*p* = 0.582). Participants were distributed across various clinical departments, demonstrating comparable distributions between the two groups. The Medical-Surgical Ward represented the largest proportion in both the study group (28.6%) and the control group (35.7%) (*p* = 0.959). Overall, no statistically significant differences were observed between groups in any baseline characteristic (all *p* > 0.05), confirming group comparability.

**Table 1:** Personal and academic data

	Variable		Control G		Study G		χ2	*p*
			No = 28	%	No = 28	%		
1	Age		The participants’ age ranged from 23 to 26 years median age 24.50 years		The participants’ age ranged from 23 to 26 years median age 24.50 years		U = 385.00	*p* = 0.902
2	Gender	Male	15	53.6	18	64.3	**χ2 =** 0.664	*p* = 0.415
		Female	13	46.4	10	35.7		
3	Level of Education	Bachelor’s	28	100	28	100	--*	--*
4	Job Title	Staff Nurse	28	100	28	100	--*	--*
5	Years of Nursing Experience		The participants’ professional nursing experience ranged from 1 to 3 years, with Median 2 year		The participants’ professional nursing experience ranged from 1 to 3 years, with Median 2 year		U = 360.50	*p* = 0.582
6	Current Department	Emergency Room (ER)	4	14.3	4	14.3	MC = 1.364	*p* = 0.959
		Hemodialysis Unit (HD)	4	14.3	2	7.1		
		Medical-Surgical Ward	8	28.6	10	35.7		
		Intensive Care Unit (ICU)	5	17.9	4	14.3		
		Coronary Care Unit (CCU)	3	10.7	4	14.3		
		Home Care Services	4	14.3	4	14.3		

χ^2^: Chi square test

MC: Monte Carlo

U: Mann-Whitney

* Statistical comparison was not applicable as the variable is constant across both groups (100%)

### Knowledge level

As for the Post-test knowledge levels in the study and control groups (Table 2), most of the participants in the study group attained high level of knowledge (42.9%) compared with the control group which had a majority of 50% who had low levels of knowledge about post-test levels. The results of the independent t-test showed a statistically significant difference between groups (t = 3.322, *p* = 0.002), and a large effect size (Cohen’s d = 0.89). ANCOVA indicated that there was a significant difference between groups (F = 25.32, *p* < 0.001).

**Table 2:** Post-test knowledge levels in the study and control groups

Post level	Control G 9.68 ± 1.82		Study G 11.61 ± 2.470		t-test	ANCOVA
**Knowledge Category**	No = 28	%	No = 28	%	t = 3.322 *p* = 0.002*	F = 25.32 *p* < 0.001
Low	14	50.0	5	17.9		
Moderate	13	46.4	11	39.3		
High	1	3.6	12	42.9		

t: independent t-test

*: Statistically significant at *p* ≤ 0.05

Within the study group, drug dose calculation knowledge improved significantly from pre- to post-intervention (Table 3). Paired t-test analysis confirmed a statistically significant improvement (t = 7.985, *p* < 0.001). At baseline, (64.3%) of participants demonstrated a low knowledge level, with no participants achieving a high level. Following the intervention, (42.9%) achieved a high knowledge level and (17.9%) remained at a low level.

**Table 3:** Comparison of pre-test and post-test knowledge scores in the study group

Pre & post study	Pre 8.36 ± 2.313		Post 11.61 ± 2.470		*p*
**Knowledge Category**	No = 28	%	No = 28	%	t = 7.985 *p* < 0.001*
Low	18	64.3	5	17.9	
Moderate	10	35.7	11	39.3	
High	0	0	12	42.9	

t: Paired-Samples t-test

*: Statistically significant at *p* ≤ 0.05

### Clinical decision making (CDM)

Post-intervention clinical decision-making scores differed significantly between groups (Table 4), (46.4%) of participants reported a moderate level of CDM regarding the control group with only (14.3%) of participants achieving a high level, while (42.9%) achieved a high level regarding the study group. There is a statistically significant difference between the study and control groups in the post-test CDM score (t = 3.597, *p* = 0.001) a large effect size observed (Cohen’s d = 0.9). ANCOVA revealed a significant difference between groups, (F = 12.35, *p* = 0.001).

**Table 4:** Comparison of post-test clinical decision-making (CDM) scores between the study and control groups

Post-CDM of study and control groups	Control G 21.29 ± 6.32		Study G 27.61 ± 6.81		t-test	ANCOVA
	No = 28	%	No = 28	%	t = 3.597 *p* = 0.001*	F = 12.35 *p* = 0.001
Low	11	39.3	4	14.3		
Moderate	13	46.4	12	42.9		
High	4	14.3	12	42.9		

t: independent t-test

*: Statistically significant at *p* ≤ 0.05

Within the study group, CDM improved significantly from pre- to post-intervention (Table 5). There is a statistically significant difference (t = 5.665, *p* < 0.001). (67.9%) of participants demonstrated a low level of CDM regarding the pre-levels. While (42.9%) reached a high level regarding the post-levels.

**Table 5:** Comparison of pre-test and post-test clinical decision-making (CDM) scores in the study group

Pre and post CDM of study group	Pre 17.96 ± 4.77		Post 27.61 ± 6.817		p
	No = 28	%	No = 28	%	t = 5.665 *p* < 0.001*
Low	19	67.9	4	14.3	
Moderate	9	32.1	12	42.9	
High	0	0	12	42.9	

t: Paired-Samples t-test

*: Statistically significant at *p* ≤ 0.05

### General self-efficacy (GSE)

Post-intervention GSE scores differed significantly between groups (Table 6), most participants (64.3%)| reported a moderate level of GSE regarding the control group and (35.7%) a low level, while (57.1%) reported a moderate level of GSE and (25%) a high level regarding the study group, a larger proportion of participants in the study group achieved a high level of GSE (25.0%) compared to in the control group, highlighting the effectiveness of the intervention. There is a statistically significant difference between the study and control (t = 3.031, *p* = 0.002) with a large effect size observed (Cohen’s d = 0.81). ANCOVA revealed a significant difference between groups, (F = 16.54, *p* < 0.001).

**Table 6:** Comparison of post-test general self-efficacy (GSE) scores between the study and control groups

Post- GSE of study and control groups	Control G 22.71 ± 3.51		Study G 26.18 ± 4.92		t-test	ANCOVA
	No = 28	%	No = 28	%	t = 3.031 p =0.002*	F = 16.54 *p* < 0.001
Low	10	35.7	5	17.9		
Moderate	18	64.3	16	57.1		
High	0	0	7	25.0		

t: independent t-test

*: Statistically significant at *p* ≤ 0.05

Within the study group, GSE improved significantly from pre- to post-intervention (Table 7) (t = 5.315, *p* < 0.001). More than half of the participants (53.6%) demonstrated a low level of GSE at the pre-test. After the intervention, (57.1%) of participants achieved moderate levels, with (25.0%) reaching a high level.

**Table 7:** Comparison of pre-test and post-test general self-efficacy (GSE) scores in the study group

Pre and post GSE of study group	Pre 20.71 ± 2.98		Post 26.18 ± 4.92		p
	No = 28	%	No = 28	%	t = 5.315 *p* < 0.001*
Low	15	53.6	5	17.9	
Moderate	13	46.4	16	57.1	
High	0	0	7	25.0	

Paired-Samples t-test

*: Statistically significant at *p* ≤ 0.05

Additional baseline and supporting analyses, including pre-test comparisons and control group outcomes, are provided in Supplementary Tables S1–S6.

### Analysis of focus group results (Inductive thematic analysis)

Four focus group discussions, each comprising seven nurses from the intervention group (*n* = 28), were conducted over 45–60 min and moderated by an experienced qualitative researcher. Sessions were audio-recorded, transcribed verbatim, and analyzed using Braun and Clarke’s thematic analysis framework. Thematic saturation was achieved by the fourth focus group, yielding six themes.

#### Theme 1: Perceived clinical benefits

Most participants reported that the training of AI based drug calculation significantly enhanced their understanding of patient cases and their ability to make safe clinical decisions.

understand the patient’s condition and saving lives (P4, FG1), providing a clearer vision of real-time clinical changes (P9, FG2)

#### Theme 2: Realism & theory-to-practice

The majority of participants reported that the simulation improved the relevance of mathematical principles in practical scenarios by successfully integrating theoretical classroom topics with clinical application.

simulating real clinical environment more effectively than traditional methods (P12, FG3), allowing nurses to apply theoretical knowledge to a real patient safely (P5, FG1)

#### Theme 3: Confidence & competence

The majority of participants reported a significant improvement in their ability to perform calculations with confidence and accuracy.

improves our ability to solve problems rapidly, accurately, and easily (P7, FG1), fostering a sense of readiness for emergency dosing (P15, FG3)

#### Theme 4: Ease of use & engagement

Participants emphasized how the learning approach and the interface of the AI based platform were described as user-friendly, successfully enabling access to a platform features.

AI-based mobile application was highly interactive, easy to follow, fun and more engaging than we expected (P21, FG4), the interface intuitive and easy to follow (P2, FG1)

#### Theme 5: Efficiency & resource saving

Participants highlighted the advantages of using AI to speed up learning and support decision-making.

“ the app is an excellent information source” (P18, FG3), “helps make right decisions and saves lots of time” (P25, FG4).

#### Theme 6: Challenges & suggestions for improvement

Although the mobile learning application was reported as highly effective, some participants highlighted that the process needed more initial help and content depth and that they briefly hesitated when switching to new technology.

briefly hesitated when switching to new technology (P11, FG2), suggested that more initial guidance could ease the digital transition (P28, FG4)

The six identified themes and their core findings are summarized in Table 8.

The adverse events or unintended effects were not reported by the participants in either the intervention or the control group during the study period.

**Table 8:** Summary table of focus group results (Inductive Thematic Analysis)

Theme	Core finding
1. Perceived Benefits	Potential for improved patient condition analysis and safety.
2. Realism	Successful bridge between theory and bedside practice.
3. Confidence	Increased speed and smoothness in solving problems.
4. Ease of Use	High engagement through a simple, interactive interface.
5. Efficiency	Potential timesaving in decision-making processes.
6. Improvement	Desire for more advanced cases and regular updates.

## Discussion

This study evaluated the effectiveness of an artificial intelligence-based mobile learning application on nursing’s drug calculation knowledge, decision-making, and self-efficacy, while exploring its feasibility and acceptability in training contexts. The observed improvement in drug dose calculation knowledge scores observed in the intervention group may reflect the educational value of adaptive AI-based learning. This result suggests that AI-based learning environments may be associated with higher cognitive engagement with drugs calculation content through the use of case-based scenarios and adaptive feedback systems. This was echoed in qualitative focus groups who reported that the realism of the scenarios assisted in bridging theoretical knowledge and clinical practice. By integrating theory and practice, these technologies assist nursing staff to build their skills and confidence prior to interacting with patients by providing practical training and immediate performance feedback. This is in line with Kolb’s experiential learning theory, the interactive nature of the AI platform may have allowed learners to consolidate theoretical knowledge through repeated, contextualized practice. The present findings are consistent with previous research where AI-based adaptive learning and AI-based chatbots significantly improved nursing medication calculation performance [25, 26]. However, others indicated that, although post-test scores have improved, they may still fall short of the proficiency needed for clinical safety, with only a small percentage of students achieving perfect scores [16, 18].

The study showed an improvement in clinical decision skills among intervention group which may be related to the interactive and scenario-based nature of AI-based training. Qualitative results unveiled potential mechanisms which may explain why the intervention positively influenced the participants’ performance and clinical decision making. For instance, initial discussions centered on perceived benefits such as better analysis of patient condition and enhanced clinical confidence, indicating how the intervention may have supported clinical decision-making. The findings of the present study are consistent with several studies which indicated that integration of AI based learning into clinical simulation provided interactive, dynamic and scenario-based experiences and adjusts simulator feedback based on nursing student’s response [27]. This technology facilitates the automatic collection and analysis of performance data, providing students with prompt, unbiased feedback to nurses [28]. This would facilitate error correction, supports best practices, and promotes critical thinking, evidence-based decision-making, and care prioritization [27, 29]. However, Höhne et al. (2025) stated that there is little evidence supporting AI-based learning for practical medical skills [30]. These inconsistent findings could be due to differences in participant characteristics, implementation contexts, theoretical foundations, and intervention design. Compared to more static methods, interventions focused on the concepts of experiential and adaptive learning may encourage higher levels of engagement and knowledge application. Furthermore, professional nurses may react differently than nursing students because they can connect learning exercises to past clinical experiences and drug administration responsibilities. Variations in reported outcomes may also be caused by differences in educational background, organizational support, and familiarity with technology. Studies showing limited benefit may have used fewer interactive platforms or focused on single-session exposures without adaptive elements, which may have limited both engagement and knowledge transfer.

Improvements in self-efficacy observed among nurses receiving AI-based learning may reflect the positive impact of consistent practice and immediate feedback mechanisms. This suggests that engaging with interactive, scenario-based AI tools may be associated with improvements in learners’ confidence in their ability to perform tasks effectively. Likewise, in focus group discussions participants reported that they could solve clinical problems more quickly and more smoothly, which they attributed to growing confidence through repeated practice. These experiences indicate that a better sense of competence and psychological preparedness may have been built by repeated successful practice in adaptive scenarios. Since repeated successful performance in adaptive scenarios may have created a sense of mastery that transferred into increased psychological readiness [31]. These findings are consistent with Bandura’s self-efficacy theory, which posits that mastery experiences provided here through repeated successful AI-based practice are the most powerful source of self-efficacy enhancement [4]. Similarly, Stake-Nilsson et al. (2022) indicate that interactive AI-based drug calculation teaching methods significantly enhance students’ self-efficacy and drug calculation skills compared to traditional methods [2]. On contrary, McMullan et al. (2011) reported non-significant differences in self-efficacy improvement between the intervention and control groups for a specific student cohort, suggesting that the effectiveness of AI-assisted or computer-assisted methods may be affected by group or context characteristics [32].

The current study revealed learners’ high levels of interest and engagement which may have been associated with realistic clinical scenarios, personalized feedback and the user-friendly interface of the developed application, which ultimately drove the robust statistical outcomes across all variables. This aligns with Joshi et al. (2026) who reported that the acceptance and efficient utilization of AI platforms in education of nursing staff, especially for drug calculation and health teaching, depend heavily on their perceived usability and user-friendly layout. Interactive and user-friendly interfaces can reduce cognitive strain and make difficult subjects more understandable for nurses with little technical or digital competence [33].

Although there are many advantages of using AI in nursing education and practice, there are several challenges with AI integration into skills and practice training. These challenges include algorithmic bias, ethics, system usability, nurses training needs and workflow integration. Therefore, it is crucial to address these issues and provide nursing students and professionals with personalized training and operation support to ensure successful implementation [34]. The present findings may have implications for resource-constraint healthcare context to develop such training programs with minimal disruption of the nursing workflow. However, implications for patient safety, cost-effectiveness, and workforce-wide scalability need further investigation. The impact of user-centred artificial intelligence design on learner’s engagement and learning outcomes in diverse clinical settings also warrants further analysis.

## Conclusion

The findings of this study indicate that an AI-based adaptive mobile application could increase knowledge, clinical decision-making skills and self-efficacy in drug-dose calculation among the sampled nurses within the context of this study. Qualitative results complemented these findings, indicating that nurses perceived greater clinical confidence, felt better prepared to apply drug-dose calculation skills in practice, and regarded the training scenarios as realistic and reflective of real clinical situations. These findings indicate that AI-driven simulation could potentially assist in the in-service training of nurses, especially in resource-constrained environments where standardized training in medication safety is most crucial; however, wider implementation requires further investigation. Future multi-site, longitudinal trials are needed to establish the potential for long-term retention and clinical impact.

## Recommendation

### Practice and education implications

Implementing AI-based learning in healthcare institutions for medical training and continuing education programs could enhance medication administration practice; therefore, institutions must be prepared, technologically equipped, and ensure that staff members possess adequate digital literacy for successful implementation. AI-driven performance analytics can be valuable for nurse managers to identify and focus on training needs for individual nurses, and for nursing educators to consider using AI-powered scenarios besides their current teaching approaches.

### Research implications

#### Long-term effectiveness

Future studies should focus on the long-term maintenance of drug calculation skills acquired with the use of AI and the direct impact of AI on medication errors in clinical practice as ongoing competency is critical to patient safety and improved clinical outcomes.

#### Implementation research

More multi-site trials with different samples would be beneficial to improve external validity and facilitate the application of AI-based learning in a variety of healthcare contexts.

#### Technological innovation

However, to further improve realism, engagement, and learning results, the potential of future technologies, such as AI-integrated virtual reality simulation, could be explored systematically.

## Limitations of the study

There are some limitations to be noted in this study, which should be considered in interpreting the results. Firstly, the relatively small sample size may have affected the statistical power and precision of the estimates of the effects; larger multicenter studies are recommended for external validity. Secondly, recruitment at one institution limits generalizability and replication in a variety of healthcare environments is warranted. Third, the follow-up period is brief, which restricts conclusions about follow-up intervention effectiveness; future research should include long-term follow-up measures to assess retention. Fourth, outcome measures collected by self-report and testing limit the direct applicability of findings in real-world clinical practice, such as medication error rates. Finally, although efforts were made to control and reduce group interactions, the hospital-based setting could not be eliminated as a possible source of performance bias from contamination between groups, or from a Hawthorne effect. However, this study followed a rigorous mixed-methods RCT design with the potential to fill the knowledge gaps in using AI-based applications in in-service medical and nursing education in resources poor settings.

## Supplementary Information

Below is the link to the electronic supplementary material.

Supplementary Material 1

Supplementary Material 2

## Data Availability

The data that support the findings of this study are available from the corresponding author upon reasonable request. The AI-powered mobile application developed for this study is also available for academic and evaluation purposes upon request, subject to intellectual property considerations.

## Code availability

The code of the mobile application that supports the findings of this study are available from the corresponding author upon reasonable request.

## Declarations

### Reporting method

The study adhered to EQUATOR guidelines: CONSORT for the randomized trial and GRAMMS for the qualitative component.

### Data acquisition and compliance

All data utilized in this manuscript were lawfully acquired. The study does not involve genetic resources as defined by the Nagoya Protocol. Ethical approval was obtained from the Ethics Committee of the Faculty of Nursing, Alexandria University, (Approval No. IRB00013620).

## Abbreviations

AI: Artificial intelligence
MCQs: Multiple choice questions
NLP: Natural language processing
VR: Virtual reality
RCT: Randomized controlled trial
ECG: Electrocardiogram
CDM: Clinical decision making
GSE: General self efficacy
CVI: Content validity index
