# Supplementary material for: Adaptive AI-based mobile simulation for drug calculation competency in nurses: a mixed-methods RCT
Source: BMC Nurs. 2026 Sep 8;25:828. doi: 10.1186/s12912-026-05313-4 (PMC13555860; doi:10.1186/s12912-026-05313-4)
Supplement: Supplementary file 1 [file 12912_2026_5313_MOESM1_ESM.docx]

**Supplementary Table S1: Pre-Test Knowledge Levels in the Study and Control Groups**

| **Pre level** | **Control G**  **8.96±1.81** | | **Study G**  **8.36± 2.31** | | **Relation** |
| --- | --- | --- | --- | --- | --- |
| **Knowledge Category** | **No=28** | **%** | **No=28** | **%** | t = 1.093  p = 0.280 |
| Low | 19 | 67.9 | 18 | 64.3 |  |
| Moderate | 9 | 32.1 | 10 | 35.7 |  |
| High | 0 | 0 | 0 | 0 |  |

t: independent t-test

**Supplementary Table S2 : Comparison of Pre-Test and Post-Test Knowledge Scores in the Control Group**

| **Pre & post Control** | **Pre**  **8.96±1.81** | | **Post**  **9.68±1.82** | | **Relation** |
| --- | --- | --- | --- | --- | --- |
| **Knowledge Category** | **No=28** | **%** | **No=28** | **%** | t = 2.585  p = 0.015 |
| Low | 27 | 96.4 | 14 | 50.0 |  |
| Moderate | 1 | 3.6 | 13 | 46.4 |  |
| High | 0 | 0 | 1 | 3.6 |  |

Paired-Samples t-test

**Supplementary Table S3: Comparison of Pre-Test Clinical Decision-Making (CDM) Scores Between the Study and Control Groups**

|  | **Study G**  **17.96 ±4.77** | | **Control G**  **16.54±1.95** | | **Pre-CDM of Study and Control Groups** |
| --- | --- | --- | --- | --- | --- |
| **t= 1.466**  **Sig =0.151** | **%** | **No=28** | **%** | **No=28** |  |
|  | 67.9 | 19 | 89.3 | 25 | Low |
|  | 32.1 | 9 | 10.7 | 3 | Moderate |
|  | 0 | 0 | 0 | 0 | High |

t: independent t-test

**Supplementary Table S4: Comparison of Pre-Test and Post-Test Clinical Decision-Making (CDM) Scores in the Control Group**

|  | **Post**  **21.29±6.32** | | **Pre**  **16.54±1.95** | | **Pre and post CDM of Control Group** |
| --- | --- | --- | --- | --- | --- |
| **t= 4.360**  **p <0 .001*** | % | No=28 | % | No=28 |  |
|  | 39.3 | 11 | 89.3 | 25 | Low |
|  | 46.4 | 13 | 10.7 | 3 | Moderate |
|  | 14.3 | 4 | 0 | 0 | High |

Paired-Samples t-test

**Supplementary Table S5: Comparison of Pre-Test General Self-Efficacy (GSE) Scores Between the Study and Control Groups**

|  | **Study G**  **20.71±2.98** | | **Control G**  **21.89±3.35** | | **Pre-GSE of Study and Control Groups** |
| --- | --- | --- | --- | --- | --- |
| **t= 1.389**  **Sig = 0.171** | % | No=28 | % | No=28 |  |
|  | 53.6 | 15 | 42.9 | 12 | Low |
|  | 46.4 | 13 | 57.1 | 16 | Moderate |
|  | 0 | 0 | 0 | 0 | High |

t: independent t-test

**Supplementary Table S6: Comparison of Pre-Test and Post-Test General Self-Efficacy (GSE) Scores in the Control Group**

|  | **Post**  **22.71±3.51** | | **Pre**  **21.89±3.35** | | **Pre and post GSE of Control Group** |
| --- | --- | --- | --- | --- | --- |
| **t= 3.481**  **Sig = 0.002** | % | No=28 | % | No=28 |  |
|  | 35.7 | 10 | 42.9 | 12 | Low |
|  | 64.3 | 18 | 57.1 | 16 | Moderate |
|  | 0 | 0 | 0 | 0 | High |

Paired-Samples t-test
