# Supplementary material for: Adaptive AI-based mobile simulation for drug calculation competency in nurses: a mixed-methods RCT
Source: BMC Nurs. 2026 Sep 8;25:828. doi: 10.1186/s12912-026-05313-4 (PMC13555860; doi:10.1186/s12912-026-05313-4)
Supplement: Supplementary file 2 [file 12912_2026_5313_MOESM2_ESM.docx]

Data collection

Analysis

Study Phases

- Randomized Controlled Trial (RCT) - Pre/Post-test (n = 54 nurses)
- Random Allocation (1:1)
- Intervention Group (AI-powered drug calculation simulation n=28)/ Control Lecture-based training Group (n=28)
- Baseline Assessment (Knowledge, CDM, Self-efficacy)
- Two weeks intervention
- Posttets Assessment

Quantitative Data Analysis (SPSS, descriptive statistics, inferential statistics, Effect sizes)

QUANTITATIVE PHASE

Purposeful sampling intervention from group (n = 28)

QUALITATIVE PHASE

Thematic Analysis (Braun & Clarke, 2006)

Focus Group Discussions (FGDs)

INTEGRATION OF FINDINGS

- Quantitative Results & Qualitative Themes
- Explanation of outcomes
- Enhanced interpretation of intervention effectiveness

Figure 2: Explanatory sequential mixed methods design diagram
